# Controversies in the Design of Strategies for the Cure of HIV Infection

**DOI:** 10.3390/pathogens12020322

**Published:** 2023-02-15

**Authors:** Alejandro de Gea-Grela, Santiago Moreno

**Affiliations:** 1 Department of Internal Medicine, Hospital Universitario La Paz, 28034 Madrid, Spain; 2 Department of Infectious Diseases, Hospital Universitario Ramón y Cajal, Instituto Ramón y Cajal de Investigaciones Sanitarias (IRYCIS), Alcalá University, 28034 Madrid, Spain; 3 CIBER de Enfermedades Infecciosas (CIBERINFEC), Instituto de Salud Carlos III, 28034 Madrid, Spain

**Keywords:** HIV, cure, eradication, latency, reservoirs, persistent replication

## Abstract

The cure for chronic human immunodeficiency virus (HIV) infections has been a goal pursued since the antiretroviral therapy that improved the clinical conditions of patients became available. However, the exclusive use of these drugs is not enough to achieve a cure, since the viral load rebounds when the treatment is discontinued, leading to disease progression. There are several theories and hypotheses about the biological foundations that prevent a cure. The main obstacle appears to be the existence of a latent viral reservoir that cannot be eliminated pharmacologically. This concept is the basis of the new strategies that seek a cure, known as kick and kill. However, there are other lines of study that recognize mechanisms of persistent viral replication in patients under effective treatment, and that would modify the current lines of research on the cure of HIV. Given the importance of these concepts, in this work, we propose to review the most recent evidence on these hypotheses, covering both the evidence that is positioned in favor and against, trying to expose what are some of the challenges that remain to be resolved in this field of research.

## 1. Introduction

Since the discovery of the human immunodeficiency virus (HIV) and after the first antiretroviral treatments (ART), the search for a definitive cure for the infection has been a priority. However, there are characteristics of the biology of the virus itself that make this strategy particularly complex. Although it was initially thought that ART could be sufficient to cure the infection, it was soon stated that when ART is discontinued, even in patients with an undetectable viral load for prolonged periods of time, there is a clear resurgence of the infection. There are several theories supported by solid studies that attempt to explain this fact. The main obstacle appears to be the establishment of a state of viral latency in certain cells, which can also explain why the circulating virus is detected, although at a very low copy number, despite successful ART. The final result has been that, despite the efforts and resources invested in research, the sterilizing cure of the disease has only occurred in very specific cases [1,2,3,4,5]. No attempt to eliminate the latent virus has shown any significant success.

However, it could be that viral latency is not the only mechanism that explains the absence of an effective cure. In this paper, we review the available evidence on theories of viral persistence, mainly viral latency and persistent replication, trying to analyze how they affect the main lines of research on HIV eradication. For that purpose, we have performed a narrative review and update on experimental studies, systematic reviews, clinical trials and theoretical reviews/seminars on the aspects related to HIV viral latency, perspectives on cure strategies and persistent viral replication.

## 2. Viral Latency and the Latent Cell Reservoir

### 2.1. What Does Latent Viral Reservoir Mean?

Latently infected cells are defined as those that carry integrated genetic material (provirus) but do not produce infective virions, and are a part of the latent viral reservoir in patients who maintain a suppressed plasma viral load [6,7].

The virus that persists, replicating in patients on ART, comes from cells that were infected before the initiation of ART [8]. In addition, cells containing the virus can proliferate even under the pressure of the antiretroviral treatment itself, regardless of the chosen regimen [9]. However, the latent virus itself is not actively transcribed, so the immune system does not recognize cells carrying such material [10].

As early as the end of the 20th century, it was hypothesized that infected CD4 T cells, even under ART, would be sufficient to establish a viral reservoir that would allow active replication if treatment were abandoned [11]. This reservoir is stable and it is not influenced by the duration of ART [12,13]. Therefore, the persistence of the viral genome, which is transcriptionally silent but competent to reactivate viral replication, is considered the main factor limiting the ability of ART to achieve a cure of the infection [14,15,16].

The latent viral reservoir has some characteristics that are worth highlighting. Firstly, the virus that has integrated genetic material and therefore remains in a state of latency does not generate an antigenic capacity that can activate an immune response [8,12,17]. The main cells that maintain the reservoir are memory CD4+ lymphocytes, and to a lesser extent, naïve CD4+ lymphocytes [15]. Cells of the myeloid series also appear to play a role in the establishment of the reservoir, mainly due to cellular interactions with memory CD4+ T cells [18,19,20,21].

The study of the size of the viral reservoir has been performed via two methods: quantitative viral outgrowth assay (QVOA) or PCR of proviral DNA. The correlation between the results obtained in both methods is not good, which adds difficulties when designing new studies [8,22]. However, QVOA has estimated that the number of cells carrying the viral reservoir is low [23,24]. Cohn et al. [23], in 2017, reported how CD4+ T cells containing latent reservoir express genes that prevent them from cell death.

Therefore, although CD4+ T cells that act as a latent viral reservoir do not actively transcribe genetic material, if these lymphocytes enter a clonal replication cycle, they give rise to new virus copies [17,23]. CD4+ T cells may undergo clonal expansion as a consequence of interactions with antigens, homeostatic proliferation or due to alterations in the expression of different genes modulated by proviral genetic material [25,26,27]. This minimal but persistent viral load prevents the cure of the disease only under ART [28,29] and it is also responsible for viral regrowth after treatment cessation [6].

Among the cells that act as a reservoir for the virus, up to 95% of them could carry defective proviruses containing genetic information that prevents them from replicating [6]. However, an estimated 7–12% of cells may contain viruses with intact infectious capacity [8,13], which could led to virological failures [25,30]. In addition, recent work by the Lichterfeld’s group supports the existence of natural selection of latent provirus in patients over time [31]. Anyway, cells containing defective viruses are able to transcribe genetic material and produce viral proteins that stimulate CD8+ T cells [32,33,34], so defective proviruses would have a relevant role in the search for curative strategies targeting the latent reservoir [35].

The time from infection to ART initiation is important in establishing the size of the viral reservoir. It has been investigated whether very early initiation of ART, less than 2 days after a primary infection, could prevent the establishment of the latent viral reservoir. While initial research in simians seemed promising [36], the available information now indicates that although patients with very early initiation of ART have a smaller viral reservoir, it does not completely prevent its establishment, and therefore early initiation of ART alone is not sufficient to achieve the cure of the disease [6,37,38,39,40,41,42]. The reservoir is established at very early stages of the disease, even in the first week after primary infection [6,8,36,39]. Lymphocytes of intestinal lymphoid tissue and lymph nodes are infected at very early stages of infection, before seroconversion and the circulation of infected cells at the plasma level [43].

Establishing the latent viral reservoir in memory CD4+ T lymphocytes provides a clear biological advantage to the virus. The virus is only detected at the plasma level during active replication and less than 2% of CD4+ cells circulate in plasma [6]. After an immune response mediated by CD4+ T lymphocytes, a minimal part of these remains latent in lymph nodes as memory CD4+ T lymphocytes. These cells, whose half-life exceeds 40 years, maintain a low level of replication and clonal re-expansion, which makes the elimination of any viral genetic material virtually impossible. Stem memory cells, a subtype of memory CD4+ T cells, seem to have a greater tendency to contain proviral DNA [44] and exhibit a great capacity for clonal expansion and to perpetuate the latent reservoir [6,45]. Follicular T-helper cells could also act as a reservoir [15] due to the absence of an interaction with CD8+ T lymphocytes, which are theorized to have a fundamental role in the immune response to HIV [46,47,48].

Thus, after a primary HIV infection, a small number of CD4+ memory T cells are infected by the virus (unlike the rest of the CD4 cells, which are rapidly destroyed after infection) and establish a reservoir. These cells remain in a quiescent state until certain antigenic stimuli lead to clonal proliferation cycles, which may in part explain the detection of detectable viral loads despite properly administered antiretroviral treatments. Cells carrying the latent viral reservoir go undetected by the immune system. The long half-life of these cells has made HIV infection incurable to date. It should be also noted that other tissues can also act as a reservoir, compromising future strategies for eliminating the virus.

### 2.2. Where Is the Viral Reservoir Located?

**Figure 1:**
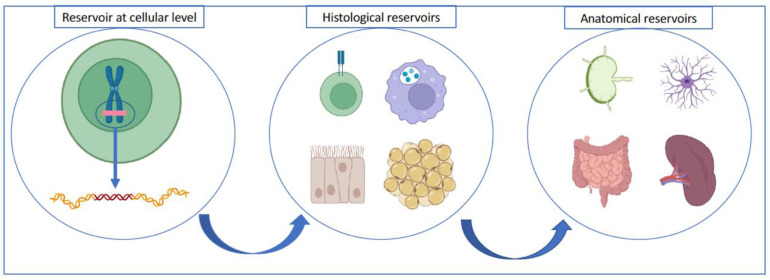
Simplified anatomical distribution of the viral reservoir: different cell lines contain viral genetic material integrated into the genome. CD4+ T cells are the most prominent, with the memory cell lineage being of particular relevance. However, other cell assemblies also act as a viral reservoir, such as dendritic cells/macrophages, epithelial cells or adipose tissue cells. In terms of anatomical distribution, the reservoir in lymph nodes stands out, especially at the level of GALT (lymphoid tissue associated with the intestine), together with other locations, such as the spleen or microglia at the level of the central nervous system [14]. Created in biorender.com (accessed on 17 October 2022).

The distribution of latently infected cells has been a matter of intense research, but is not well understood at the moment. In 2019, Vibholm et al. [49] found that proviral genetic material was similar in latent cells isolated from plasma and in lymph nodes, but was different from that isolated when patients stopped ART. Those facts supports that the latent reservoir is stable and constitutes the basis on which the virus replicates when pharmacological pressure ends. Other cells of the immune system such as follicular dendritic cells, or even fibrocytes or epithelial cells could also act as a reservoir [50]. The role of monocytic series cells as cells that contain provirus interacting with CD4+ cells, has been previously discussed [20,21,51,52,53] (Figure 1).

Different tissues can act as a reservoir, although not all have been well studied or characterized [24]. Yukl et al. [54] showed that the viral reservoir in intestinal tissue, which is usually overexposed to infectious agents, had different characteristics from those in other organs, largely due to the greater proportion of effector memory T cells. In addition, other immune system cells present in intestinal tissue also contained genetic material. These characteristics make intestinal tissue an ideal site for the establishment of the viral reservoir.

### 2.3. Implications of Clonal Replication in Viral Reservoirs

**Figure 2:**
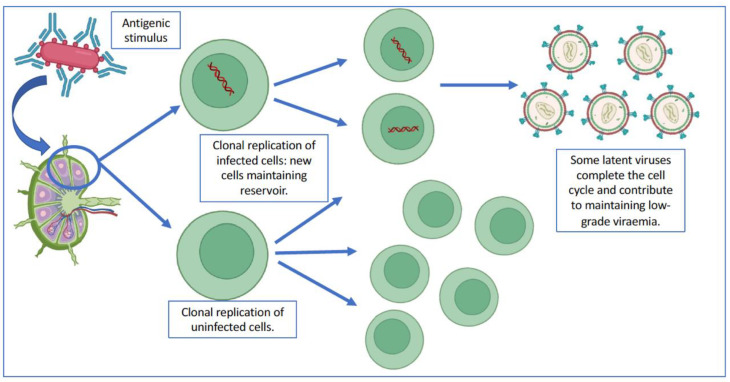
Clonal replication in the latent viral reservoir in lymph nodes. Following an antigenic stimulus, or simply following a homeostatic replication cycle, cells containing integrated genetic material would enter into viral replication cycles, which contributes to maintaining the latent viral reservoir and would be the cause of persistent viremia despite ART. Created in biorender.com (accessed on 17 October 2022).

Halvas et al. [55] tested several of the concepts described above. Eight patients with a persistently detectable viral load, despite correctly ART administered for more than 6 months, were selected. All patients had a viral load in the low-level viraemia range, which has been classically associated with an increased risk of virologic failure [56,57,58]. Comparative analysis of viral genetic material in different samples showed that most of the copies detected came from the same viral clone. No resistance development was detected in the study. These data reinforced the hypothesis that the clonal expansion of previously infected cells is a cause of detectable plasma viral load, simulating virological failure (Figure 2).

The authors also pointed out that if the permanent viral reservoir is a cause of virologic failure, despite adequate ART, it would be interesting to find a drug that acts by blocking virion production. However, since these antivirals are not currently available, it is appropriate to pursue better treatments to seek a cure for the disease, provided that the clonal replication of infected cells may be a clear barrier in the search for a cure for the disease.

### 2.4. Towards a Cure for HIV Infection Based on the Elimination of the Latent Reservoir

One of the main strategies in the search for a cure for HIV is the “shock and kill” method. It is based on achieving the reversal of virus latency in infected cells, so that these viruses are “reactivated” and the cells containing them are no longer hidden from the immune system [24]. In a second step immune response, inducing agents would be administered to pursue a cure.

In the first phase of the shock and kill strategy, latency-reversing agents (LRAs) are administered in order to reverse the latent state of the virus [59,60]. Then, the elimination of infected CD4+ cells is promoted, either by innate immune responses or by the administration of specific drugs. However, LRAs still do not have a decisive influence on reducing the size of the viral reservoir [61].

The first trials based on this strategy date back to the beginning of the current century. Interleukin-2 (IL-2) and anti-CD3 antibodies were investigated as LRA, but a lack of efficacy as well as adverse effects stopped the use of these agents [61,62]. Subsequently, other drugs such as disulfiram or valproic acid were investigated [59,63]. However, the most promising drugs come from the field of oncohematology: vorinostat (treatment of cutaneous lymphomas), panobinostat (treatment of multiple myeloma), ipilimumab (treatment of melanoma) and romidepsin (treatment of T-cell lymphomas) have all been investigated in recent years as promising LRAs [64,65]. Additionally, drug combinations such as disulfiram and romidepsin have been studied [66].

However, these drugs fail to eliminate the latent reservoir for several reasons. Rasmussen et al. [61] pointed to immunological factors, such as an inadequate immune response by cytotoxic CD8+ T cells or the fact that memory CD4+ T cells do not necessarily die when the virus is reactivated. However, two other very relevant factors should be emphasized. When an LRA is administered, there is an increase in plasma viral load, but we are unable to quantify the percentage of the reservoir that has been mobilized and, therefore, the real effect of the drugs on the reservoir. Furthermore, a large majority of the viral reservoir is found in tissues such as the intestinal tract or lymph nodes. Given that the pharmacokinetics in these compartments vary with respect to the plasma compartment, it cannot be assured that the latent reservoir in these tissues will be reached by LRAs.

Therefore, the strategy of reversal of viral latency seems to be effective in that it manages to stimulate the exit of latency in the virus from the lymphocyte reservoir [67,68] but the reservoir at other anatomical levels seems to remain intact [69]. Estes et al. studied lymphoid tissues in both human and primate models [70]. Under ART pressure, most of the reservoir consisted of intestinal lymphoid tissue (GALT) cells, and these cells were largely responsible for viral load regrowth upon the cessation of ART. The strategy of latency reversal by means of LRA must take into account the existence of other compartments that were not well reached by the drugs mentioned above [71,72,73].

Telwatte et al. [74] analyzed how anti CD3/CD28 drugs can be more effective in the reversion of the intestinal reservoir. The previously mentioned LRAs appear not to be as effective in acting on this anatomical reservoir, while the combination of ingenol mebutate with romidepsin appears to be more effective than other regimens. Moreover, given the increasingly well-established relationship between HIV infection and the persistent proinflammatory state, the search for drugs that cover the largest possible proportion of the viral reservoir have become a priority [75]. There is research into new molecular signaling pathways to improve the latency reversal process [76,77,78], although the complex signaling process regulating CD4+ T cell expression is not yet fully understood [79].

However, even if a latency-reversing drug could overcome all the above obstacles, the most suitable kill strategies would still have to be established [80]. Skepticism about the shock and kill strategy is promoted by data such as those from the RIVER clinical trial, in which there were no significant differences in terms of total HIV DNA between the ART group and the ART + LRA + immune response stimulator drug group [81]. It is true that the interpretation of the results of the trial may be influenced by the fact that the variation in total HIV DNA does not correctly reflect the percentage of the reservoir attacked or the potency of the immune response generated.

Even so, immune stimulatory drugs fail to coordinate a cytotoxic cellular response that effectively eliminates the affected cells [82]. In addition to a lack of recognition of the signals expressed by the infected cell [83,84,85], it appears that certain ARLs such as romidepsin may even interfere with the intensity of the immune response [86]. Even so, hopes are still pinned on the search for new signaling pathways on which to act: since the discovery of the PDL-1 signaling pathway [87], progress has been made in discovering cellular receptors such as TIM-3, whose blockade can enhance the immune response to the virus [88]. In addition, active efforts are being directed towards the search for a drug that combines the necessary properties to unify the action of both shock and kill (“All in one”) [82].

The block and lock strategy is one of the most novel ones and consists of a totally opposite approach to shock and kill. Block and lock is based on preventing the reactivation of the latent reservoir by inhibiting viral RNA expression even under stimulatory signals [89]. Latency-inducing drugs are used [90] with the intention of maintaining the virus in a permanent state of latency, even after the definitive abandonment of ART [91,92]. Agents useful for this strategy, named latency-promoting agents, could affect both pre-integration and post-integration stages. LEDGINs are inhibitors of the lens epithelium-derived growth factor (LEDGF/p75) that can redirect HIV DNA integration to regions that are resistant to reactivation, thus silencing the reservoir even after ART discontinuation [93]. At post-integration stages, drugs can be used to permanently suppress HIV-1 transcription and prevent viral reactivation even after successful proviral DNA integration. This aim can be achieved by inhibiting viral and host transcription factors to suppress viral gene expression. Some examples include didehydro-cortistatin A (dCA) which inhibits Tat-mediated transactivation [94], CDK9 inhibitors which block viral transcription by disrupting P-TEFb formation [95], or curaxin which inhibits NF-kB [96].

Based on these same concepts, the idea of using gene-editing technology, such as the CRISPR-CAS system, was born [97]. This technique would seek to modify the genetic transcription systems by means of “molecular scissors”, maintaining the entire reservoir in a permanent state of latency.

In any case, the current outlook does not envisage a definitive cure for HIV, at least in the short term. All the approaches described are based on the fact that the main cause of the lack of effective strategies for a cure is the failure to reach the entire viral reservoir. It is worth recalling the complexity of estimating its size using current molecular detection techniques [98]. However, there could be indications that the virus persists in infected patients not only because of latency, but also because there are anatomical sanctuaries in which there is sustained viral replication, and which are not reached by adequate concentrations of antiretroviral drugs. In this scenario, any strategy based exclusively on the reversal of viral latency could risk failure, since it would ignore the existence of other compartments that perpetuate viral persistence. Therefore, the next topic to be discussed will be to delve into the various studies that have analyzed the possibility of sustained viral replication in various anatomical locations, and the consequences that this could entail.

## 3. Persistent Viral Replication

### 3.1. Is There Persistent Viral Replication in Patients with a Suppressed Plasma Viral Load?

The previous sections have reviewed the evidence on viral latency, which explains, in part, the impossibility of curing HIV infection by antiretroviral treatment alone, so the possibility of other therapeutic routes has been explored. However, at the beginning of the 21st century, several experiments were carried out with children infected at birth, in whom plasma viral load was still detected despite appropriate treatment. Since the isolated viruses did not contain sequences confirming a resistance to antiretrovirals, it was interpreted that this situation was caused by the existence of an established reservoir [99,100,101]. However, in recent years, other complementary and not exclusive explanations to this model have been proposed.

**Figure 3:**
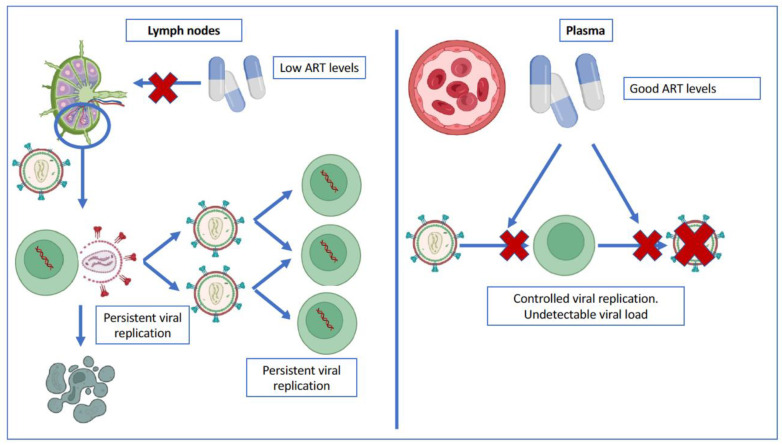
Persistent viral replication in lymph nodes. Adequate levels of ART in the vascular compartment block viral replication, thus maintaining undetectable plasma viral loads. However, at the same time, ART does not reach adequate levels in lymph nodes, so there are complete cycles of viral replication and new cell infections at this level. Created in biorender.com (accessed on 17 October 2022).

One such explanation would be that the virus may be actively replicating continuously in another anatomical compartment such as lymphoid tissue. This idea is mainly supported by the work carried out by Lorenzo-Redondo et al. in 2016 [102]. They analyzed the viral sequences isolated in patients after 3 and 6 months from the initiation of ART, and pointed out that the sequences were different when comparing isolates from lymph nodes with those from the plasma compartment. Therefore, the authors proposed that the virus is distributed in two separate compartments, with very little exchange of viral particles between them. A first compartment corresponds to the plasma space, where drug concentrations are high and viral replication would be controlled. On the other hand, a compartment composed of anatomical sanctuaries, such as the lymphoid tissue [103], would have little drug penetration, which would allow a low but persistent level of viral replication (Figure 3). Two subpopulations of the virus would coexist in the lymph node: a majority of the population of infected cells would be in a quiescent state, which would replicate irregularly but sufficiently to maintain persistently positive viral loads (cells that maintain the virus in a state of latency, as already discussed in previous sections), and another subpopulation of cells in which the virus replicates persistently, despite treatment.

### 3.2. Evidence in Favor of Persistent Viral Replication and Its Causes

Persistent viral replication would imply that the virus could have very low level of replication localized in lymph nodes, despite ART. Subsequently, this virus could also be the determinant of the residual viremia detected in plasma [104]. Rose et al. attempted to demonstrate why that viremia could be explained by persistent replication in the lymph nodes [105,106]. There is also evidence from autopsies of chronically infected patients with an undetectable plasma viral load that the virus persists in different tissues [107], especially at the level of the central nervous system [3,108] and lymphoid and adipose tissues [109].

Thus, the theory of persistent viral replication in anatomical sanctuaries has raised several important debates. First, Licht and Alter [110] offered important reflections: If the drugs did not penetrate the lymph nodes, could this fact be responsible for persistent viral replication and could it induce the development of ART resistance? Lorenzo-Redondo [102] supported that persistent replication in lymphoid tissue was responsible, at least in part, for the residual viral load [104]. However, despite the subtherapeutic levels that drugs reach in lymph nodes, no resistant mutants are selected [111]. Lorenzo-Redondo explained this fact by means of mathematical models, in which, when effective drug concentrations are low, the benefit of drug resistance does not overcome the fitness cost of mutations and drug-sensitive strains dominate.

There are other studies that support those hypothesis: Fletcher [112] investigated the deficient pharmacokinetic properties of antiretroviral drugs when reaching certain anatomical locations and Estes [70] detected intracellular virions in animal models, despite ART, using in situ hybridization techniques.

Supported by Litch and Alter’s definition of lymph nodes as immunological and pharmacological sanctuaries, pharmacokinetic studies that analyze the tissue penetration of antiretroviral drugs have emerged [112,113,114]. A significant proportion of drug penetration studies in lymph nodes have been performed in animal models, and human models have shown heterogeneous data [115], although derived data supports the limited penetration of ART in these compartments.

Using mathematical models, Jagarapu et al. [116] determined which factors condition the lower levels of antiretroviral drugs at the lymph node level. They mainly emphasized that the larger diameter of the lymph node, which translates into inflammation, is the main factor limiting the distribution of drugs in this compartment. They determined that in lymph nodes with diameters less than 0.1 mm (which translates to an absence of inflammation) there is no persistent viral replication despite low drug levels, although the chances of replication increase as the node size increases. Protease inhibitors [117,118] are the therapeutic group that diffuses the worst in this tissue, and regimens based on these drugs are the ones that could be most associated with the establishment of drug sanctuaries [116]. Tenofovir disoproxil difumarate, emtricitabine, atazanavir, darunavir and efavirenz also have poor activity in lymphoid tissue [112,118], as well as integrase inhibitors [118,119,120,121]. However, there is recent evidence supporting the better diffusion of tenofovir alafenamide at the lymph node level, which would be an theoretical advantage when using these drugs [122] (Table 1).

Therefore, if penetration into the lymph nodes could be improved, therapeutic levels could be achieved that could control viral replication at this level. This opens the door to research on new lines of administration, such as targeted delivery strategies, in which the drug is coformulated with molecules with a high affinity for certain tissues (as in the case of chylomicrons and lymphatics) [123,124,125,126].

### 3.3. Controversies on Persistent Viral Replication

Shortly after the publication of the work analyzed by Lorenzo-Redondo [102], several groups of authors began to defend the inexistence of persistent viral replication. Rosenbloom et al. [127] argued that the data collected by Lorenzo-Redondo were obtained from patients after 3 and 6 months of ART, at which time there is still a significant proportion of labile viral subpopulations, given that the permanent viral reservoir is not fully established until 12 months. Therefore, they argue that the viral sequences isolated and interpreted as a consequence of persistent viral replication were nothing more than a phenomenon typical of the early stages of the natural evolution of the virus after the initiation of ART. They also reflect that, in the case of persistent viral replication, it would be important to know what clinical repercussions it would have, adding that a degree of subcritical replication would not have an impact on HIV cure strategies [128].

In the same way, Kearney et al. [129] proposed alternative hypotheses to explain the results observed by Lorenzo-Redondo. They used as an argument, among others, that the genomic variability of the sequences of the viruses isolated a few months after the onset of ART could also correspond to proviruses mutated under cycles of clonal expansion.

For this, they rely on the theory of the fossil record phenomenon, which assumes that, in the first months after ART initiation, experimentally detectable viral genomic sequences may reflect that their origin comes from viral replication, but this is only an “artifact”, which will disappear when the fossil record is completely “washed out” after 12 months of ART [130]. Therefore, they again criticize the fact that results were obtained earlier than 12 months after the start of ART, assuming that the results of Lorenzo-Redondo’s work would have been different if the samples had been taken much later. They add to these assertions with new mathematical simulations, similar to those provided in the previously discussed publications [127,129].

In several subsequent papers, this group of authors reaffirm that persistent viremias are the exclusive consequence of the clonal replications of latently infected cells [55,131,132,133,134]. They highlight several key aspects: firstly, viruses isolated as a consequence of the clonal replication of cells with integrated viral genetic material do not express drug resistance; furthermore, these clones are established very early despite the rapid onset of ART [135] and are responsible for the latent viral reservoir that prevents the cure of the disease [136].

Controversies also reach studies on cellular models in which the point of integration of the proviral genome is evaluated. In this sense, there is evidence in favor of persistent viral replication, while Symons et al. found that there were different sites of genomic integration in cellular models of latent infection, suggesting active replication [137]. However, in mathematical models [26,130], stability in the genomic integration sites has been evidenced, which supports the hypothesis of clonal expansion as the only mechanism of viral persistence.

### 3.4. Potential Consequences of Persistent Viral Replication in Patients with Suppressed Plasma Viral Load

The existence of persistent viral replication despite ART would imply a new source of viremia that cannot be controlled with the ART regimens currently used [138]. In this regard, intensification regimens have been tested, based on the theory that a higher drug load could help to better control replication and decrease residual viremia. However, the results have been heterogeneous [138,139], with evidence both in favor of pharmacological intensification helping to decrease immunoactivation levels [140,141,142], and recent studies opposing this idea [143,144].

Of particular importance is the state of persistent inflammation, which has recently been related to various adverse effects in the long term, such as cardiovascular diseases or neoplasms [145,146]. In this sense, there are multiple lines of research currently seeking to analyze strategies to reduce the state of immunoactivation [147], for example, by controlling the microbiota [148], but undoubtedly, the existence of persistent viral replication could be a key factor in modulating the level of inflammation.

Finally, assuming the existence of persistent viral replication would open the door to reconsidering the role of bitherapy as ART, since according to the positions of some different authors, it could determine a negative role in the control of markers of persistent inflammation in the long term [149]. However, other groups argue that there would be no significant differences in terms of inflammation parameters when comparing triple with dual therapies [150].

In any case, the debate about the existence or not of persistent viral replication, despite the maintenance of suppressed plasma viral loads, is a debate that has not been closed at present, and whose future implications may be relevant to finding a definitive cure for HIV infection.

## 4. Conclusions: Where Do We Stand on a HIV Cure?

Current strategies are not sufficient to achieve a cure for HIV infection. The persistence of a latent viral reservoir in patients with suppressed plasma viral loads in certain cellular and anatomical locations means that, with repeated cycles of clonal re-expansion, low-level viraemia is maintained, which justifies a resurgence of the viral load as soon as the antiretroviral treatment is abandoned. Current virus elimination strategies using kick and kill techniques appear to be promising, but have limitations when it comes to knowing and reaching the entire viral reservoir, which is essential for eventual complete virus elimination. In this context, study groups have emerged advocating the existence of persistent viral replication cycles despite antiretroviral treatment. Although these assertions have aroused great controversy in recent years, the implications for understanding the biology and behavior of the viral reservoir would be fundamental when focusing on new strategies in the search for a cure for the infection. In this work we have focused on reviewing the current evidence on these aspects, trying to emphasize the current fields in which new studies are needed, and trying to clarify what may be alternative future lines of research.

## Figures and Tables

**Table 1:** Summary of factors contributing to low or adequate penetration of antiretroviral drugs into lymph nodes [116,117,118,119,120,121,122]. ^1^ Not currently available.

Low Levels	Appropriate Levels
Thickness of the diameter of the ganglion wall greater than 0.1 mm.	Absence of inflammation at the lymph node level.
Use of integrase inhibitors.	Use of tenofovir alafenamide
Use of protease inhibitors.	Targeted delivery and nanoparticle technologies ^1^
